# Isolation and identification of the spoilage microorganisms in Sichuan homemade Paocai and their impact on quality and safety

**DOI:** 10.1002/fsn3.1148

**Published:** 2019-08-05

**Authors:** Xiaolin Ao, Junling Yan, Ceng Chen, Jiawei Zhao, Shuliang Liu, Ke Zhao, Shujuan Chen, Li He

**Affiliations:** ^1^ Sichuan Agricultural University Yaan China

**Keywords:** identification, isolation, quality analysis, Sichuan Paocai, spoilage microorganism

## Abstract

The spoilage microbiology in Paocai (fermented vegetables) affects not only the quality of this popular traditional Chinese food but also its safety. This study aimed to isolate and identify the microorganisms commonly leading to spoilage in homemade Paocai from the Sichuan region and, further, to scientifically assess the impact of these microorganisms on product quality and safety. Seven putrid Paocai samples were collected from 7 families in different Sichuan cities. From these, 45 strains were isolated by means of a nutrient agar medium and rose bengal agar. All of the 22 strains (5 fungi and 17 bacteria) with different colonial morphologies and corruption phenomenon in Paocai were determined 16S rDNA/18S rDNA gene sequences. Bacteria were identified as *Bacillus* spp. (16 strains) and *Staphylococcus saprophyticus* (1 strain), while the 5 fungi were identified as *Candida* spp. (3 strains), *Kodamaea ohmeri* (1 strain), and *Geotrichum candidum* (1 strain). Based on the results of identification, 7 representative strains of different species were determined as the spoilage characteristics in paocai. All the representative strains metabolized to produce nitrite. Strain SPF21 (*K. ohmeri*) has a particularly serious impact on the crunchiness of Paocai; however, strains SPF19 (*Bacillus subtilis*) and SPF21 have the greatest influence on its color. All five of the fungi were seen to form a film on the surface of the Paocai, with SPF5 (*G. candidum*) exerting the most extreme influence. The growth characteristics in the broth showed that all the representative strains investigated metabolized most of the carbohydrates and were able to tolerate the salinity and acidity of the ordinary homemade Paocai. Moreover, these strains were found to have obvious impacts on the volatile components of Paocai, especially SPF2 and SPF8, with higher dimethyl disulfide and dimethyl trisulfide, which were found to have a pungent odor when highly concentrated.

## 1 INTRODUCTION

Paocai, or fermented vegetable, has a history of thousands of years in China, and Sichuan Paocai is considered to be the most famous of these traditional recipes (Zhang et al., 2016). Paocai is a brine‐salted pickle, fermented mainly by naturally adherent microorganisms on the vegetable, especially lactic acid bacteria (LAB). The raw material in Paocai is crisp and tender vegetables, such as radish, cabbage, mustard, and cowpea. Paocai is often served as an appetizer in the traditional Chinese diet. Its uniquely appetizing features include sourness, a distinct aroma, freshness, and crispness, while its nutritional advantages include strengthening the spleen, promoting digestion, antioxidation, antibacteria, lowering cholesterol, prevention hypertension, and tumors, all of which explains the popularity of this dish (Seong‐Eun et al., 2019; Xia et al., 2017). However, Paocai is usually homemade by Sichuan families all year round, so the success of the fermentation process tends to be dependent on the indigenous microflora adhering to the vegetables. Moreover, the quality is affected by factors such as the environment and the changing seasons, which results in numerous uncertainties in the process and outcome (Xiong, Guan, Song, Hao, & Xie, 2012). When either the temperature or the aerobic environment is unfit for the growth of LAB, spoilage microorganisms flourish, causing the corruption of Paocai in terms of flavor, biofilms on the surface, color deterioration, and harmful compositions, which severely affect the quality, edibility, and safety of the Paocai.

Previous research has reported *Bacillus* spp., yeast, and aerogenic LAB as the dominant spoilage microorganisms in fermented vegetables (Wang, Zhang, Chen, & Zhang, 2018; Wang et al., 2015; Zhang et al., 2018). The reproduction and metabolism of the spoilage microorganisms greatly influence the quality of Paocai for the following three reasons: first, their metabolites negatively affect the flavor and sensory quality; second, some microorganisms can produce toxins in the metabolic process that seriously affect the safety of products; and, finally, some microorganisms can convert nitrate into nitrite through metabolic processes, thereby increasing the risk of cancer. Therefore, the purpose of this study is to provide theoretical basis for improving the quality and safety control of homemade Paocai by determining the species of spoilage microorganisms and their influences on the quality of Paocai.

## 2 MATERIALS AND METHODS

### 2.1 Sample collection

Samples of Paocai were collected from seven families in seven different cities in Sichuan, in July, 2017, the season in which Paocai is most prone to corruption. The samples were numbered A‐G, respectively, and transported to the laboratory in an icebox for immediate analysis. The spoilage phenomena and collection information with respect to the Paocai samples were shown in Table 1.

**Table 1:** The features of the spoiled Paocai samples and the isolates

Sample	Pickled vegetable	Sampling site	Description of typical corruption phenomenon					pH value	Strain number
			Film on the face	Turbidity of brine	Color of pickle	Crunchiness	Odor		
A	Lotus root	Meishan	Sheet‐like	Turbide	Normal	Soft	Normal	3.7	SPF1 ~ SPF4
B	Ginger	Yaan	Sheet‐like	Clear	Normal	Soft	Pungent acidity	3.4	SPF5 ~ SPF9
C	Leaf mustard	Chengdu	No	Clear	Brownness	Normal	Pungent odor	3.9	SPF10 ~ SPF16
D	Radish	Zigong	No	Clear	Brownness	Normal	Pungent odor	3.5	SPF17 ~ SPF25
E	Cowpea	Meishan	White mucus	Clear	Whitening	Normal	Sour and foul odor	3.7	SPF26 ~ SPF36
F	Leaf mustard	Yaan	No	Turbide	Normal	Soft	Pungent acidity	3.9	SPF37 ~ SPF40
G	Assorted vegetables	Xichang	Sheet‐like	Turbide	Brownness	Soft	Pungent odor	3.6	SPF41 ~ SPF45

### 2.2 Isolation of spoilage microorganisms from samples

The 7 Paocai samples were diluted in different gradients and cultivated on nutrient agar media (peptone 10 g, beef extract 3 g, NaCl 5 g, agar 15–20 g, distilled water 1,000 ml) and rose bengal agar (peptone 5 g, glucose 10 g, KH_2_PO_4_ 1 g, MgSO_4_·7H_2_O 0.5 g, agar 20 g, 1/3,000 bengal red solution 100 ml, distilled water 1,000 ml, chloramphenicol 0.1 g) to isolate bacteria at 37°C for 24 hr and fungi at 25°C for 48 hr, respectively. The isolated strains were further purified and stored in 15% glycerol solution at −70°C for subsequent experiments.

### 2.3 Paocai manufacture and inoculation of tested strains

Summer radish is the most commonly used ingredient in Paocai. In this assay, 200‐g‐fresh summer radish was washed and dried on the surface, cut into strips of 10 mm × 10 mm × 80 mm, and placed in a fermentation tank, to which was added the same weight of brine (salt content 8%) (Zhou, Liu, & You, 2018)**.** The suspicious spoilage microorganisms were inoculated with 10^5^ cfu/g samples, while one uninoculated sample was used as a control. Prepared samples were sealed and fermented at 25°C for 7 days. Strains showing obvious spoilage were screened for subsequent experiments.

### 2.4 Identification of the spoilage microorganisms

#### 2.4.1 DNA extraction

Strains were harvested for DNA extraction at 37°C for 18 hr. Total bacterial DNA and fungal DNA were extracted using the Bacterial Genomic DNA Extraction kit (Tiangen Co. Ltd.) and the fungal DNA kit (OMEGA Engineering), respectively. DNA concentration and quality were determined spectrophotometrically at 260 and 280 nm. (Agilent Co. Ltd.).

#### 2.4.2 16S rDNA sequence analysis

The 16S rDNA sequences of the isolated bacteria were amplified using the primers P1 (8–27f: 5′‐AGAGTTTGATCCTGGCTCAG‐3′) and P2 (1520–1541r:5′‐AAGGAGGTGATCCA GCCGC A‐3′), and the 18S rDNA of the isolated fungi were amplified using the primers EF‐3: 5′‐TCCTCTAAATGACCAAGTTTG‐3 and EF‐4: 5′‐GGAAGGGATGTATTTATTAG‐3′ (Wilmotte, Auwera, & Wachter, 1993).

**Figure 1:**
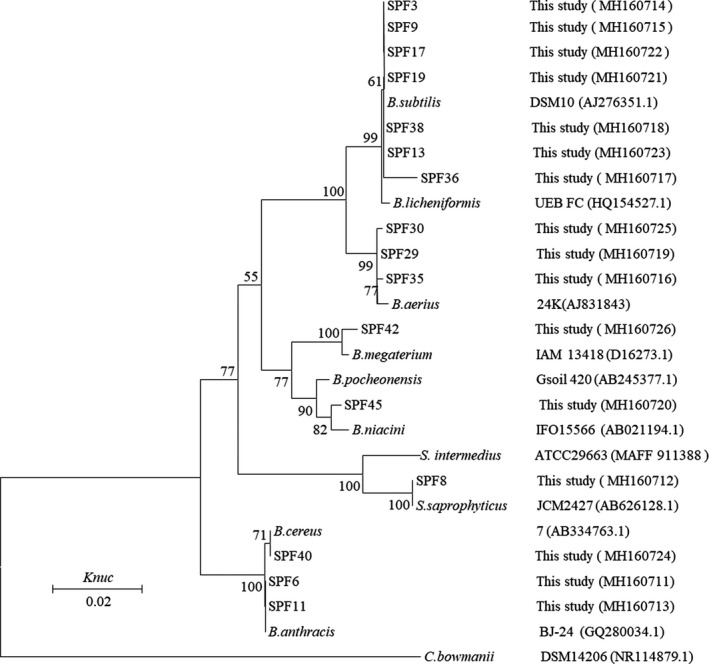
Phylogenetic tree showing the relative position of representative bacteria strains isolated from spoilage Paocai samples based on the 16S rDNA sequences. Bootstrap values (1,000 replicates) are indicated at branch nodes. *C. bowmanii* was used as an out‐group. *B.* = *Bacillus*; *S.* = *Streptococcus*; *C.* = *Clostridium*; *Knuc* = nucleotide substitution rate

**Figure 2:**
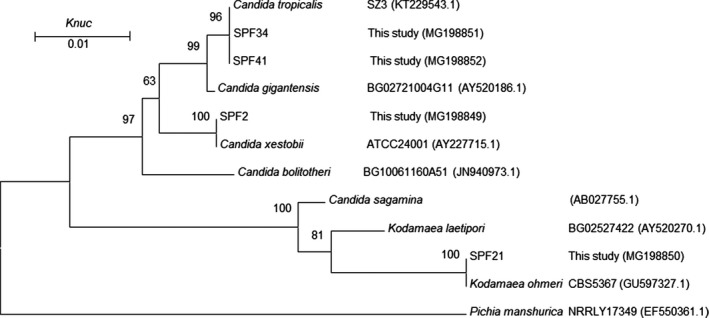
Phylogenetic tree showing the relative position of representative fungi strains isolated from spoilage Paocai samples based on the 18S rDNA sequences. Bootstrap values (1,000 replicates) are indicated at branch nodes. *Pichia manshurica* was used as an out‐group. *Knuc* = nucleotide substitution rate

**Figure 3:**
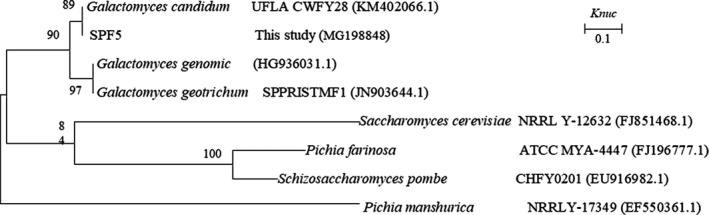
Phylogenetic tree showing the relative position of SPF5 based on the 18S rDNA sequences. Bootstrap values (1,000 replicates) are indicated at branch nodes. *Pichia manshurica* was used as an out‐group. *Knuc* = nucleotide substitution rate

The 16S rDNA and 18S rDNA genes were sequenced using an ABI 3730 sequencer (Applied Biosystems). The nucleotide sequence data were deposited in the GenBank nucleotide sequence database. The accession numbers are shown in Figures 1, 2, 3. Multiple sequences were assembled and aligned with the Clustal W program (Thompson, Cronin, & Martin, 2010). Type strain sequences for comparison were blasted against the GenBank database. The phylogenetic trees were constructed using MEGA 5.1 under the Kimura two‐parameter model and bootstrapped with 1,000 replicates.

### 2.5 Spoilage characteristics of representative strains in pickled summer radish

Based on the results of identification, the representative strains of different species were determined as the spoilage characteristics in paocai. The method of Paocai manufacture and inoculation of representative strains was described in Section 2.3.

#### 2.5.1 Physical and chemical analysis of Paocai

The chromatic aberration of the samples was measured by means of a colorimeter (Beijing Kang Guang Instrument Co. Ltd.), and the values of L*, a*, and b* were, respectively, recorded. The crunchiness of the samples was determined by means of a TA‐XT plus Texture Analyzer (Lotan Science Co. Ltd.). The samples were cut into blocks measuring 10 mm × 10 mm × 4 mm. The probe was P/5, while the test mode was compression test force, the operation started for return, the pretest speed was 5 mm/s, the measured speed was 2.00 mm/s, the post‐test speed was 10 mm/s, and the displacement was 5 mm (Kamat, Dash, & Balasubramaniam, 2018). Each Paocai sample (summer radish 5 g + brine 5 g) was crushed and filtered with three layers of gauze, after which the pH value of the sample was measured using a pH meter (Nanjing Century Ark Analytical Instrument Co. Ltd). The content of nitrite in the sample was determined using the N‐(1‐naphthyl)‐ethylenediamine dihydrochloride spectrophotometric method (Ding et al., 2018).

#### 2.5.2 Analysis of flavor components by SPME‐GC‐MS

Each Paocai sample (summer radish 2.5 g + brine 2.5 g) was treated at 40°C for 30 min using a water bath after crushing. The volatile compounds were subsequently extracted for 30 min with a manual solid phase microextraction (SPME) device equipped with a 50/30 μm DVB/CAR fiber (Supelco). After extraction, the fiber was inserted into the GC injection port and the analytes desorbed for 5 min at 250°C.

Gas chromatography–mass spectrometry (GC–MS; Agilent‐7890A‐5975C; Agilent) with an elastic capillary vessel column (HP‐5MS, 30 m × 0.25 mm, 0.25 μm film thickness) was used to analyze the volatile flavor compounds. The specific parameters were as described by Shen, Cheng, and Pu (2018).

The data were qualitatively analyzed by references and matching results, which compared with the standard mass spectrometry provided by NIST.11. The relative content of the volatile components was determined by the area normalization method.

#### 2.5.3 Growth and carbohydrate metabolism characteristics of spoilage strains in broth

The growth of the spoilage strains at different temperatures (15, 25, 35 and 45°C) was observed after incubation in nutrient broth or potato dextrose agar for 7 days. Growth at various pH (3.0, 4.0, 5.0, 6.0, and 7.0) and salt concentrates (0%, 2%, 4%, 6%, 8%, and 10%) were detected after incubation at 30°C for 7 days (Yang, Cao, Cai, & Terada, 2010). Glucose, fructose, sucrose, and starch were used as the sole carbon sources for testing the metabolism of carbon.

### 2.6 Data analysis

Data used in physical and chemical analysis were subjected to an analysis of variance, and Tukey's test was used to identify the differences (*p* < .05) between means using the GLM procedure of SAS (SAS Institute, Inc.).

## 3 RESULTS AND DISCUSSION

### 3.1 Collection of putrid Paocai samples and their isolates

The samples of putrid Paocai collected in Sichuan, as described above, showed typical spoilage phenomena, such as, biofilm or a white stickiness on the surface, turbid brine, soft and rotten, browned or faded, or unpleasant smell. The pH of the Paocai samples was measured between 3.4 and 3.9, which is the normal pH range for Paocai. Forty‐five strains were isolated from the Paocai samples and numbered SPF1‐SPF45. Information regarding the samples and isolates is shown in Table 1.

According to standards for domestic trade industry of the republic of China (SB/T 10756‐2012), Paocai is characterized by bright colors, crisp, and tender texture, and a sour but refreshing taste. However, because the raw material cannot be sterilized, vestigial spoilage microorganisms commonly compete with LAB at both the early and late stages of fermentation, causing corruption of the Paocai. This analysis of the growth features and corruption characteristics of the spoilage microorganisms is, therefore, of great significance in assisting the control of quality and safety of Sichuan homemade Paocai.

### 3.2 Identification of suspicious spoilage microorganisms

Five fungi strains and 17 bacteria were screened from 45 strains, according to their different colonial morphologies and corruption phenomenon in Paocai. The screened strains were sequenced, and the phylogenetic trees of the suspicious spoilage strains are shown in Figures 1, 2, 3.

Figure 1 shows a phylogenetic tree based on the 16S rDNA gene sequences of the isolated bacteria. The 17 strains were divided into 7 species. Strains SPF3, SPF9, SPF13, SPF17, SPF19, SPF36, and SPF38 were determined to be related to *Bacillus subtilis*. Strains SPF29, SPF30, and SPF35 were clustered together and identified as *Bacillus aerius*, while strains SPF42, SPF45, SPF8, and SPF40 were related to *Bacillus megaterium*, *Bacillus niacini*, *Staphylococcus saprophyticus*, and *Bacillus cereus*, respectively. In addition, strains SPF6 and SPF11 were related to *Bacillus anthracis*. According to the results, with the exception of SPF8, belonging to *Staphylococcus* spp., all other bacteria were *Bacillus* spp., indicating that *B. subtilis* was the dominant spoilage bacteria in the Paocai samples.

This concurs with the findings of Zhang et al., who studied the characteristics of dominant spoilage microorganisms in Paocai samples of different salinities and similarly determined that *Bacillus* spp. was the main spoilage strain, especially *B. subtilis*, which can cause turbidity, rancidity, and film formation (Zhang et al., 2018).

In the current study, the fungi in the spoiled pickles were divided into 3 species (Figure 2). Strains SPF34 and SPF41 were found to be related to *Candida tropicalis* and identified as *C. tropicalis*. In addition, strains SPF2 and SPF21 were related to *Candida xestobii* and *Kodamaea ohmeri*. In all, the results showed that five fungi, belonging to two genera, along with *Candida* spp. were the main spoilage microorganisms. Wang et al. also found *C. tropicalis* to be the main spoilage microorganism responsible for the formation of a film on the surface of Paocai (Wang et al., 2015).

As strain SPF5 was far from the other fungi in the phylogenetic tree, its result was not shown and, therefore, an individual phylogenetic tree was built for its identification (Figure 3). The strain SPF5 was related to *Galactomyces candidum* and identified as *G. candidum*. Arroyo‐López et al. reported *G. candidum* as dominant in spoiled Spanish fermented table olives, causing an obvious film and other spoilage phenomena, such as turbid brine, bad odor, or softening of the vegetable (Arroyo‐López et al., 2012)**.**

### 3.3 Effect of the spoilage organisms on the quality of Paocai

The seven strains impacting most negatively on the quality of the Paocai samples were screened, as shown in Table 2. The results showed that each of the strains had different effects on the quality of the pickle. Nitrite is an important factor in evaluating the food safety of Paocai, as the higher the nitrite content, the lower the edibility and safety of the food. According to the industrial standards for light industry of the People's Republic of China (QB/T2743‐2015), nitrite content may not exceed 2.0 mg/kg in pickled vegetables. Compared with the control, the nitrite content in all the Paocai inoculated with spoilage bacteria was significantly increased, especially with strain SPF42 (*B. megaterium*), in which the nitrite content reached up to 14.03 mg/kg. This result confirms that when Paocai is spoiled with *B. megaterium*, the security risks are increased, making the food dangerous for consumption.

**Table 2:** The effect of the screened strains on the quality of ped summer radish

	Species	Nitrite (mg/kg)	pH value	Brittleness (g)	Value of chromatism^a^			Film formation^b^
					L	a	b	
Control		0.94 ± 0.02^g^	3.69 ± 0.08^bc^	2,689.52 ± 32.30^b^	51.55 ± 1.66^c^	28.60 ± 0.63^a^	12.25 ± 0.3^c^	−
SPF‐2	*Candida xestobii*	4.22 ± 0.04^b^	3.50 ± 0.07^cd^	2,947.00 ± 0.25^a^	37.09 ± 0.59^e^	25.79 ± 1.04^b^	13.22 ± 0.35^c^	++
SPF‐5	*Geotrichum candidum*	0.78 ± 0.04^h^	3.16 ± 0.06^e^	2,015.00 ± 3.59^e^	46.51 ± 2.01^d^	28.05 ± 0.98^a^	15.17 ± 0.45^b^	++
SPF‐8	*Staphylococcus saprophyticus*	3.12 ± 0.03^c^	3.65 ± 0.24^bc^	2,542.80 ± 5.28^c^	55.43 ± 1.86^b^	8.92 ± 0.34^e^	20.68 ± 0.90^a^	−
SPF‐19	*Bacillus subtilis*	2.14 ± 0.06^d^	3.83 ± 0.13^b^	2,999.05 ± 108.45^a^	50.73 ± 0.44^c^	8.71 ± 0.22^e^	20.97 ± 1.00^a^	
SPF‐21	*Kodamaea ohmeri*	2.06 ± 0.05^e^	3.65 ± 0.21^bc^	1,346.79 ± 17.58^f^	62.87 ± 3.91^a^	7.92 ± 0.15^e^	21.47 ± 0.87^a^	−
SPF‐34	*Candida tropicalis*	1.02 ± 0.02^f^	3.36 ± 0.04^de^	2,082.81 ± 8.72^e^	55.26 ± 0.21^b^	24.28 ± 0.71^c^	12.54 ± 0.21^c^	+
SPF‐42	*Bacillus megaterium*	14.03 ± 0.05^a^	4.48 ± 0.04^a^	2,359.27 ± 20.93^d^	61.48 ± 1.13^a^	11.97 ± 0.66^d^	20.45 ± 0.36^a^	+

Note

Means with different lowercase letters in the same column differ significantly at *p* < .05.

a a: L for the lightness; a for the green‐red color; b for the blue‐yellow color.

b b: − = no film; + = thin film; ++ = thick film.

In terms of pH value, the Paocai inoculated with strain SPF42 had the highest pH value, at 4.48, while the pH value of the sample with strain SPF5 was lowest (pH 3.16). In general, the range of pH value in Paocai is 3.5–4.0. When the raw materials are the same, the differences arise mainly from the microorganisms present in the vegetables. If the acidity of Paocai cannot be effectively managed, either LAB is not the dominant microorganism and the growth of spoilage bacteria is inhibiting its growth and acid production, or the spoilage bacteria is producing alkaline substances, such as NH_3_.

Crunchiness is another important index of the quality of Paocai. The crunchier the vegetable is, the better the mouthfeel of the Paocai taste. Here, the crunchiness of the Paocai inoculated with strain SPF21 (*K. ohmeri*) was found to be lowest, at 1,342.73 g, followed by those with strains SPF5 and SPF34, all of which were significantly less crunchy than the control. The main factors affecting Paocai crunchiness are the pH value, salt, and enzymes. The spoilage microorganisms can produce pectinase, cellulase, and amylase, which all reduce crunchiness. Pectin substances, which can be decomposed by yeast and mold, and degraded sugars and acids, also significantly impact upon the crunchiness of pickles (Hernández, Martín, Aranda, Pérez‐Nevado, & Córdoba, 2007)**.** Previous reports have shown that *G. candidum* has the ability to produce pectinase, which may explain it impact on the crunchiness (Yu, Huang, & Du, 2018); however, *K. ohmeri* has not been confirmed as a cause of Paocai spoilage.

Vegetable color, such as the red of summer radish, is another indication of Paocai quality. As shown in Table 2, the greater the value L and the smaller the value b, the better the quality of the Paocai. The results showed that the L values of the samples inoculated with isolates were lower than those in the control, thus indicating that different strains have a certain effect on darkening the Paocai. Strain SPF2 (*C. xestobii*) was found to significantly affect the brightness of the raw material. The values of a and b indicate that strains SPF8, SPF19, and SPF21 discolored the pickled radish from red to yellow and blue, indicating that these spoilage organisms have a significant effect on the color of Paocai.

Moreover, the formation of a biofilm on the surface of brine is an obvious spoilage phenomenon, always accompanied by a deterioration in taste and smell. From the results, strains SPF5 and SPF21 formed the most serious biofilms, while the biofilm effect of SPF34 was also obvious. If these microorganisms exist in Paocai, a biofilm will occur when LAB is not dominant.

### 3.4 Physicochemical characteristics of the spoilage organisms

The seven screened strains were cultured in liquid media under different conditions. The results are shown in Table 3. Most strains grew optimally at 25–45°C, with the exception of SPF8, which could not grow at 45°C, while strains SPF5, SPF19, and SPF34 could not grow at 15°C. These results explain why Paocai is more likely to rot in summer and autumn, when daily temperatures average between 25 and 45°C. The results of the pH range indicate that strains SPF5 and SPF2 grow at a relatively narrow pH range of 4–6, which is the acidity at the beginning of Paocai fermentation. Therefore, such strains are generally growing in competition with LAB at the beginning of fermentation, sometimes causing the process to fail. However, the strains SPF34 and SPF42 can grow in a wide pH value range of 3–7, so, even when the paocai ferments normally and the acidity is reduced, the growth of such microorganisms cannot be inhibited when the conditions are suitable. The introduction of oxygen and oil, for example, or a rise in temperature will assist in the rapid multiplication of these microorganisms, causing decomposition of the Paocai.

**Table 3:** Physicochemical characteristics of screened strains

	Temperature range (°C)	pH range	Salt concentrate range (%)	Glucose	Fructose	Sucrose	Starch
SPF‐2	15–45	4–5	0–6	+	−	+	+
SPF‐5	25–45	4–6	0–4	+	+	+	+
SPF‐8	15–37	5–7	0–10	−	−	+	−
SPF‐19	25–45	5–7	0–5	+	−	+	+
SPF‐21	15–45	5–7	0–6	+	−	+	+
SPF‐34	25–45	3–7	0–5	+	−	+	−
SPF‐42	15–45	3–7	0–5	+	−	+	−

All the tested strains were found to tolerate salt concentrations ranging from 4% to 6%, which is the same concentration commonly used in homemade Paocai. In order to inhibit the growth of the spoilage microorganism, the salt concentration is sometimes increased to 10%, which makes the paocai taste especially salty and has a harmful effect on health. Strain SPF8 (*S. saprophyticus*) can grow at a salt concentration of 10% and, therefore, even some Paocai with high salt levels are at risk of being contaminated by this species (Pérez‐Díaz et al., 2019).

The pickled vegetables are comprised mainly of glucose, fructose, sucrose, and starch. The results of this study showed that all the spoilage strains can metabolize glucose and fructose to produce an acidic substance. The exceptions to this are SPF8, which cannot metabolize glucose, SPF5, which cannot metabolize fructose or starch, and strains SPF2, SPF19, and SPF21, none of which are able to metabolize starch. The more carbon a microorganism uses, the stronger its ability to grow competitively. Therefore, SPF5 (*G. candidum*), SPF19 (*B. subtilis*), and SPF21 (*K. ohmeri*) are most likely to compete with LAB for growth, resulting in pickle corruption.

### 3.5 Effects of spoilage microorganisms on volatile components of Paocai

Paocai is famous for its sour fragrance and flavor. The most important flavor components in Sichuan Paocai are dimethyl sulfides and aldehydes, among others (Zhang et al., 2018)**.** The various effects of different spoilage bacteria strains on the flavor components in the Paocai samples in this study are listed in Table 4. In pickled summer radish, the main flavor substances were found to be isothiocyanates, sulfur compounds, siloxane, and aldehydes. Isothiocyanates and sulfocompounds are characteristic flavor substances in radishes and were noted in all samples.

**Table 4:** Volatile components of Paocai inoculated with screened strains

No.	Components	Formula	CK	SPF‐2	SPF‐5	SPF‐8	SPF‐19	SPF‐21	SPF‐34	SPF‐42
			(%)							
1	4‐Methylpentyl isothiocyanate	C_8_H_7_NOS	5.34	5.06	—	2.41	3.84	2.47	3.56	—
2	Butane, 1‐isothiocyanato‐	C_5_H_9_NS	2.34	—	—	16.5	—	7.96	6.24	0.36
3	Hexane, 1‐isothiocyanato‐	C_7_H_13_NS	—	2.52	0.94	3.23	1.14	—	4.23	—
4	Hexamethylcyclotrisiloxane	C_6_H_18_O_3_Si_3_	5.04	2.28	1.22	1.06	2.66	0.9	1.36	1.37
5	Dimethylcyclosiloxane	C_8_H_24_O_4_Si_4_	3.36	2.81	—	0.79	2.77	0.82	0.94	—
6	Dodecamethylcyclohexasiloxane	C_12_H_36_O_6_Si_6_	2.05	1.25	0.82	0.65	1.48	0.56	0.76	1.75
7	Tetradecamethylcycloheptasiloxane	C_14_H_56_O_7_Si_7_	2.25	0.8	0.32	0.32	0.99	0.31	0.37	1.35
8	Dimethyl disulfide	C_2_H_6_S_2_	24.17	51.56	28.19	28.03	24.64	22.96	40.33	25.92
9	Dimethyl trisulfide	C_2_H_6_S_3_	4.98	20.25	17.06	32.41	13.8	46.59	3.4	26.95
10	3‐Thietanol	C_3_H_6_OS	—	—	—	—	—	—	13.91	—
11	Hexanal	C_6_H_12_O	2.23	—	3.11	—	—	—	—	1.45
12	1‐Nonanal	C_9_H_18_O	4.62	1.93	3.35	1.06	1.99	0.83	1.83	2.13
13	Decanal	C_10_H_20_O	—	0.86	1.37	—	0.27	—	—	0.24
14	Linalool	C_10_H_18_O	12.2	—	14.67	4.48	14.43	4.95	11.1	11.47
15	d‐Limonene	C_10_H_16_	6.21	0.61	4.45	0.38	—	0.37	0.56	5.71
16	p‐Cymene	C_10_H_14_	—	—	—	0.25	—	—	—	0.37
17	p‐mentha‐1,5‐diene	C_10_H_16_	1.52	—	—	0.18	—	—	—	—
19	Tetrasulfide, dimethyl	C_2_H_6_S_4_	—	—	—	3.16	1.13	5.15	—	1.73
22	2‐Pentyl furan	C_9_H_14_O	—	4.31	2.79	—	1.54	—	—	—
	Total		76.31	94.24	78.29	94.91	70.68	93.87	88.59	80.8

Compared with the control, the concentration of isothiocyanates was found to be very low in the samples after inoculation with strains SPF5 and SPF42, at only 0.94% and 0.36%, respectively, thus indicating that the material can be decomposed by these strains, thereby reducing the radish flavor. Conversely, isothiocyanate was higher in the samples than in the control level after inoculation with strains SPF8 and SPF21, at 16.5% and 7.96%, respectively, the effect of which produced a pungent raw radish flavor in the Paocai.

Similarly, all samples contained dimethyl disulfide and dimethyl trisulfide; however, the concentrations were higher in the samples inoculated with spoilage bacteria than in the control, especially those with strains SPF2 and SPF8, which are the main flavor substances in many Paocai products. Zheng, Zheng, and Li (2007) and Yoon et al. (2008) determined the flavor substance in Korean pickle and also found sulfur compounds, including dimethyl disulfide and dimethyl trisulfide, to be the main compounds (Yoon et al., 2008; Zheng et al., 2007). The flavor of a sulfur substance can be experienced at a low threshold (0.16 µg/kg), therefore, when the content is too high, it will produce a typical irritant smell, which will seriously affect the overall flavor and taste of Paocai. Moon, Chang, Kim, and Chang (2014) reported that sulfur compounds, including dimethyl disulfide and dimethyl trisulfide, deteriorate the flavor of kimchi (Moon et al., 2014). Furthermore, significantly high dimethyl trisulfide content, which is consistent with this experiment, obviously softens Paocai (Zhao et al., 2016). Notably, strain SPF34 contains 3‐thietanol, which becomes foul smelling and toxic when the relative content is high. In this study, the content of 3‐thietanol reached 13.91%, thus confirming that contamination with SPF34 will affect the flavor and safety of Paocai.

In addition, all samples were found to contain aldehydes and siloxane, although these substances were less concentrated in the samples inoculated with spoilage bacteria than in the control. The above results indicate that spoilage microorganisms can degrade or inhibit flavor components in Paocai and alter the sensory perception of the food.

## 4 CONCLUSIONS

The 22 strains of spoilage microorganisms were obtained by isolation and morphological screening from seven putrefied Paocai samples from seven different Sichuan cities. Through 16S rDNA/18S rDNA gene sequencing, the main microorganisms in the samples were determined as *B. subtilis*, *C. tropicalis,* and *G. candidum*, and further tie‐back experiments confirmed which of these were responsible for the corruption of Paocai. The corruption phenomena included the production of harmful substances, such as sulfur compounds (dimethyl trisulfide), nitrite, and 3‐thietanol, causing spoilage characteristics such as malodor, film production, softness, and browning. In the process of Paocai fermentation, the microbial species are complex and the fermentation flavor is diverse. Therefore, necessary measures, such as ensuring clean raw material and an anaerobic fermentation environment, or the addition of antibacterial spice, should be taken to avoid contamination from corrupt microorganisms and ensure the safety and flavor of Paocai.

## CONFLICT OF INTEREST

The authors declare no conflict of interest or relationship, financial, or otherwise.
